# Supplementary material for: The Structural Rule Distinguishing a Superfold: A Case Study of Ferredoxin Fold and the Reverse Ferredoxin Fold
Source: Molecules. 2022 May 31;27(11):3547. doi: 10.3390/molecules27113547 (PMC9181952; doi:10.3390/molecules27113547)
Supplement: Supplementary file 1 [file molecules-27-03547-s001.zip › molecules-1678492-supplementary.pdf]

Supplementary Materials for
The structural rule distinguishing a superfold: A case study of ferredoxin-like fold and the reverse ferredoxin-like fold

Takumi Nishina, Megumi Nakajima, Masaki Sasai and George Chikenji

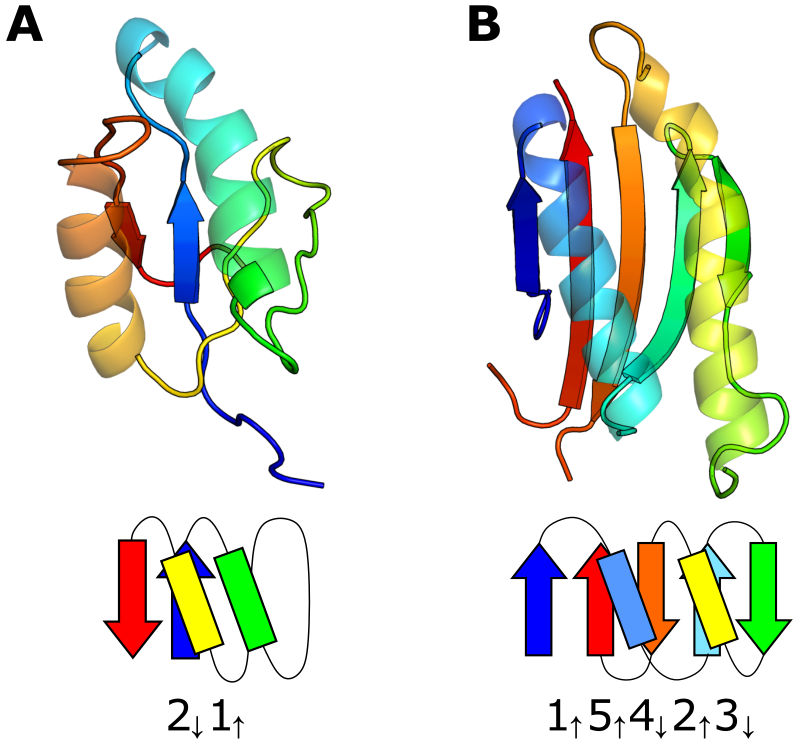

Figure S1: Examples of protein structures whose topology cannot be classified in terms of X-groups in ECOD. (**A**) Tetracycline resistance protein, tetM (PDB ID: 3J25), which belongs to the X-group representing the ferredoxin fold, but STRIDE identifies only two β-strands in tetM. (**B**) S-layer protein (PDB ID: 3CVZ), which belongs to the X-group representing the reverse ferredoxin fold, but STRIDE identifies a topology $1_{\uparrow}5_{\uparrow}4_{\downarrow}2_{\uparrow}3_{\downarrow}$ for this protein.

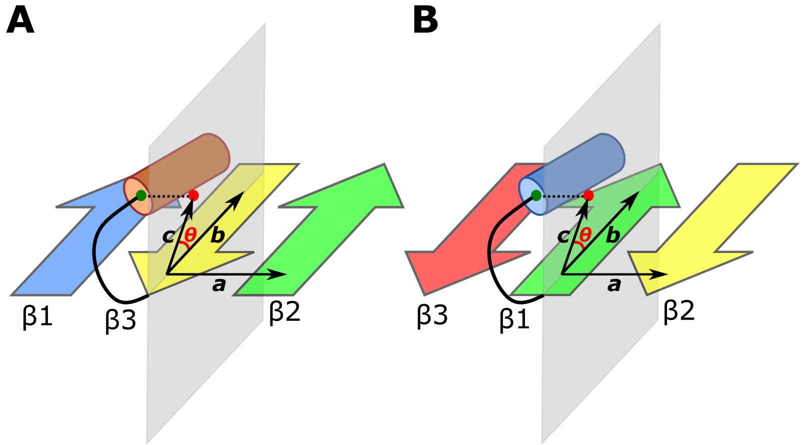

Figure S2: Definition of the angle θ to locate the position of the terminal α-helix in the $1_{\uparrow}3_{\downarrow}2_{\uparrow}+$ C-term α structure and the $3_{\downarrow}1_{\uparrow}2_{\downarrow}+$N-term α structure. When $\theta > 0$, the terminal helix and the helix in the $\beta\alpha\beta$ structure lie on the same side of the β-sheet plane, while these helices lie on the opposite side of the plane when $\theta < 0$. We defined three vectors, **a**, **b**, and **c**, and defined θ as the angle between **b** and **c**. (**A**) In the $1_{\uparrow}3_{\downarrow}2_{\uparrow}+$C-term α structure, the vector **a** is a vector extending from the Cα atom of the C-terminal residue of the β-strand 3 (yellow arrow) to the Cα atom of the N-terminal residue of the β-strand 2 (green arrow). The vector **b** is a vector extending from the Cα atom of the C-terminal residue of the β-strand 3 to the Cα atom of the second residue before the C-terminal residue of the β-strand 3. We projected the center of mass (green dot) of Cα atoms of four N-terminal residues of the α-helix (orange cylinder) onto the plane spanned by **b** and **a**×**b** (red dot). The vector **c** is a vector extending from the Cα atom of the C-terminal residue of the β-strand 3 to the projected point. (**B**) In the $3_{\downarrow}1_{\uparrow}2_{\downarrow}+$N-term α structure, the vector **a** is a vector extending from the Cα atom of the N-terminal residue of the β-strand 1 (green arrow) to the Cα atom of the C-terminal residue of the β-strand 2 (yellow arrow). The vector **b** is a vector extending from the Cα atom of the N-terminal residue of the β-strand 1 to the Cα atom of the second residue after the N-terminal residue of the β-strand 1. We projected the center of mass (green dot) of Cα atoms of four C-terminal residues of the α-helix (blue cylinder) onto the plane spanned by **b** and **a**×**b** (red dot). The vector **c** is a vector extending from the Cα atom of the C-terminal residue of the β-strand 3 to the projected point.

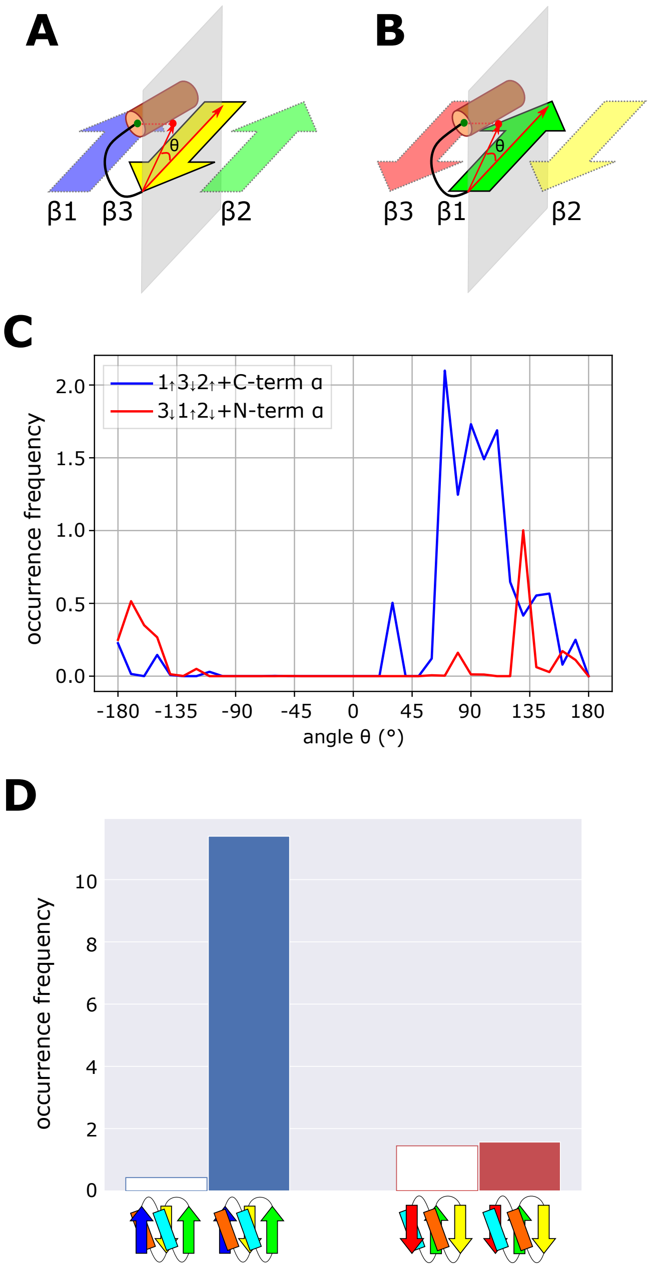

Figure S3: Occurrence frequency of θ in the dataset. θ is predominantly positive in $1_\uparrow 3_\downarrow 2_\uparrow$ + C-term α, while the occurrence frequency for $\theta < 0$ is small both in $1_\uparrow 3_\downarrow 2_\uparrow$ + C-term α and $3_\downarrow 1_\uparrow 2_\downarrow$ + N-term α. Therefore, the structure with $\theta > 0$ should explain the major difference between $1_\uparrow 3_\downarrow 2_\uparrow$ + C-term α and $3_\downarrow 1_\uparrow 2_\downarrow$ + N-term α. (**A**) θ in the $1_\uparrow 3_\downarrow 2_\uparrow$ + C-term α structure. (**B**) θ in the $3_\downarrow 1_\uparrow 2_\downarrow$ + N-term α structure. (**C**) Occurrence frequency of θ in the $1_\uparrow 3_\downarrow 2_\uparrow$ + C-term α (blue) and $3_\uparrow 1_\downarrow 2_\uparrow$ + N-term α (red) structures. The terminal helix and the helix in the $\beta\alpha\beta$ structure are on the same side of the β-sheet plane when $\theta > 0$, while they are on the opposite side when $\theta < 0$. The 99% sequence identity representatives derived from the ECOD database was used for this analysis. (**D**) Occurrence frequencies of helices being on the opposite versus the same side of the $1_\uparrow 3_\downarrow 2_\uparrow$ + C-term α (Left) and $3_\downarrow 1_\uparrow 2_\downarrow$ + N-term α structure (Right). A white bar surrounded by blue lines, a blue bar, a white bar surrounded by red lines, and a red bar represent occurrence frequencies of the $1_\uparrow 3_\downarrow 2_\uparrow$ + C-term α structures with helices being on opposite side ($\theta < 0$), those with helices being on the same side ($\theta > 0$), the $3_\downarrow 1_\uparrow 2_\downarrow$ + N-term α structures with helices being on the opposite side ($\theta < 0$), and those with helices being on the same side ($\theta > 0$), respectively.

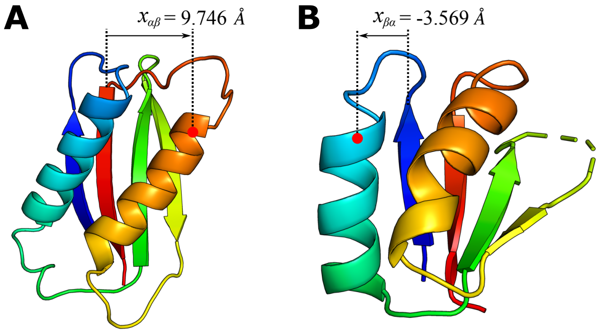

Figure S4: Examples of proteins with the reverse ferredoxin fold showing uncommon $\alpha\beta$- or $\beta\alpha$-configuration. (**A**) The catalytic core of human DNA polymerase kappa (PDB ID: 1T94) has a long $\alpha\beta$-loop showing the large $x_{\alpha\beta}$. (**B**) D-alanine–D-alanine ligase from a bacterium (PDB ID: 4EG0) shows $x_{\beta\alpha} < 0$

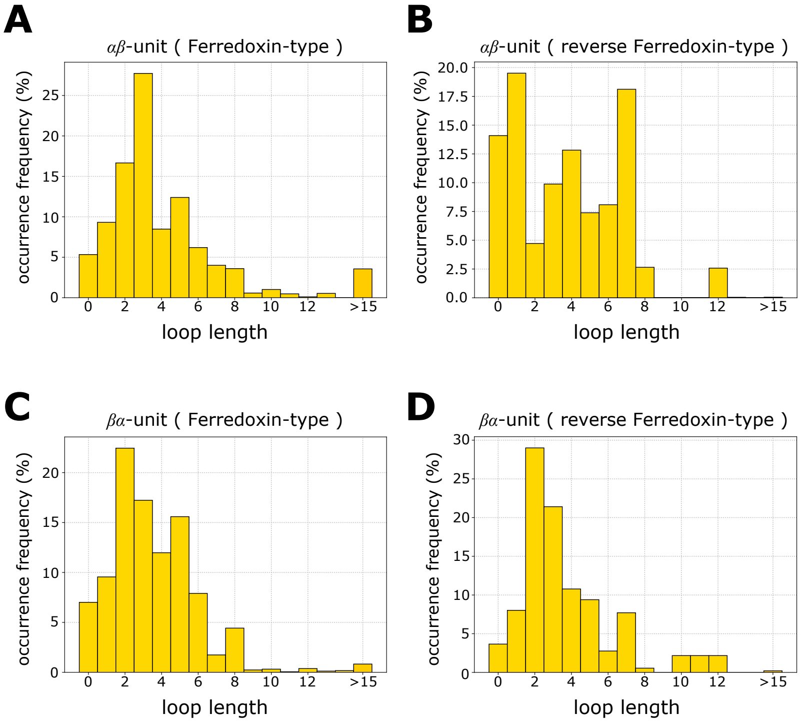

Figure S5: Distributions of loop length in the $\alpha\beta$-unit or $\beta\alpha$-unit. The loop length is defined by the number of residues constituting the loop. (**A**) The distribution of the loop in the $\alpha\beta$-unit in the ferredoxin fold. (**B**) The distribution of the loop in the $\alpha\beta$-unit in the reverse ferredoxin fold. (**C**) The distribution of the loop in the $\beta\alpha$-unit in the ferredoxin fold. (**D**) The distribution of the loop in the $\beta\alpha$-unit in the reverse ferredoxin fold.
